# Nicotinamide Riboside Alleviates the Neurotoxic Injury of Dendritic Spine Plasticity Mediated by Hypoxic Microglial Activation

**DOI:** 10.3390/biom15101391

**Published:** 2025-09-30

**Authors:** Jinchao Hou, Haowei Zhang, Xiaodong Huo, Ruili Guan, Boxuan Wang, Yuchen Wang, Fang Zhao, Xinqin Liu, Yang Hu, Congcong Zhuang, Yuankang Zou

**Affiliations:** 1 Department of Occupational and Environmental Health, The Ministry of Education Key Lab of Hazard Assessment and Control in Special Operational Environment, Fourth Military Medical University, Xi’an 710032, China; houjinchao10@163.com (J.H.);; 2 The Shaanxi Provincial Key Laboratory of Environmental Health Hazard Assessment and Protection, School of Public Health, Fourth Military Medical University, Xi’an 710032, China; 3 Cadet Regiment, School of Basic Medical Sciences, Fourth Military Medical University, Xi’an 710032, China

**Keywords:** nicotinamide riboside, hypoxia, microglial activation, neurons, synaptic plasticity

## Abstract

Exposure to hypoxia at high altitudes is significantly associated with impairments in learning and memory functions, as well as abnormalities in neuronal function and synaptic plasticity. Recent research has indicated that mitochondrial reactive oxygen species (mtROS) play a role in regulating microglial activation and mediating neurotoxic damage in the hippocampal CA1 region. Nicotinamide riboside (NR), upon absorption, is rapidly converted into nicotinamide adenine dinucleotide (NAD+), which is involved in the production of mitochondrial adenosine triphosphate (ATP). The potential of NR to protect dendritic spine plasticity in hippocampal CA1 neurons following hypoxia exposure, potentially through the inhibition of microglial activation, warrants further investigation. To this end, a mouse model simulating hypoxia at an altitude of 6000 m over a two-week period, along with a BV2 cells and conditional co-culture of BV2 cells and HT22 cells 1%O_2_ hypoxia model, was developed. Behavioral assessments indicated that, relative to the normoxia group, mice subjected to hypoxia exhibited a significant reduction in the time spent in the target quadrant, the distance traveled within the target quadrant, the number of platform crossings, and the novel object recognition index. Furthermore, Golgi staining revealed a marked decrease in the density of dendritic spines in the hippocampal CA1 region in the hypoxia-exposed mice compared to the normoxia group. Subsequently, A daily dosage of 400 mg/kg of NR was administered for two weeks and 0.5 mM NR was used in a conditional co-culture model. Results demonstrated that, in comparison to the hypoxia group, the group receiving combined hypoxia and NR treatment showed significant improvements in the time spent in the target quadrant, the distance traveled within the target quadrant, the number of platform crossings, the novel object recognition index, and the density of dendritic spines in the hippocampal CA1 region. Additionally, transmission electron microscopy indicated a significant increase in the synaptic density of hippocampal neurons in the combined hypoxia exposure and NR treatment group compared to the hypoxia exposure group. Simultaneously, when compared to the hypoxia group, the combination of hypoxia and NR treatment resulted in an increased concentration of mitochondrial ATP. This treatment also partially restored mitochondrial membrane integrity, reduced mtROS levels, decreased the percent of Iba1^+^CD68^+^Iba1^+^ microglia, and lowered the *interleukin-1β* (*IL-1β*), *interleukin-6* (*IL-6*), *tumor necrosis factor-α* (*TNFα*), and *inducible nitric oxide synthase* (*iNOS*) mRNA levels. These findings indicate that NR treatment may mitigate neurotoxic damage in the hippocampal CA1 region induced by hypoxia exposure, primarily through the attenuation of microglial activation and the reduction in mtROS production.

## 1. Introduction

With the swift resurgence of the global economy, cross-regional economic integration has become increasingly evident. An increasing number of individuals from lowland regions are migrating to or traveling within high-altitude areas. Nevertheless, the unique geographical and natural environment of plateaus—marked by complex and variable factors such as hypoxia and intense ultraviolet radiation—can adversely affect human physiological functions through multiple pathways. Among these factors, hypoxia exerts the most profound impact on the organism. Epidemiological evidence suggests a strong association between exposure to high-altitude hypoxia and the onset and progression of cardiovascular and cerebrovascular diseases [1,2,3]. Moreover, prolonged habitation in hypoxic high-altitude environments can significantly impair cognitive functions, such as judgment and reasoning [4,5,6]. Laboratory studies have shown that exposure to high-altitude hypoxia adversely affects learning and memory in mice [6,7,8]. Behavioral assessments indicate a notable increase in escape platform latency and a reduction in novel object recognition indices in these subjects [8]. Synapses, which are essential for signal transmission between neurons or between neurons and effector cells, undergo structural and numerical changes—known as synaptic plasticity [9]—that are closely associated with higher-order brain dysfunction [10]. A growing body of evidence suggests that impaired synaptic plasticity is a fundamental mechanism underlying cognitive deficits [10]. However, existing clinical treatments aimed at enhancing neuronal synaptic plasticity are hindered by limited efficacy. Therefore, further research is necessary to clarify the specific mechanisms by which hypoxia impairs synaptic plasticity and to identify potential pharmacological targets.

Hypoxia can compromise neuronal synaptic plasticity through various mechanisms, including neuroinflammation, dysfunction of mitochondrial energy metabolism, oxidative stress, and excitotoxicity linked to ionic homeostasis imbalance, ultimately leading to cognitive dysfunction. Research has shown that in several neurodegenerative disorders—such as Alzheimer’s disease (AD), Parkinson’s disease (PD), and Amyotrophic Lateral Sclerosis (ALS)—neuroinflammation directly disrupts synaptic homeostasis [11,12,13]. Evidence suggests that microglia, as the resident immune cells of the central nervous system (CNS), modulate neural circuits and higher-order brain functions through spatial heterogeneity in neuron-microglia interactions under physiological conditions [14,15]. Microglia-derived factors, such as brain-derived neurotrophic factor (BDNF), play a role in regulating both functional and structural synaptic plasticity [16,17,18,19]. Upon exposure to stressors within the CNS, microglia rapidly become activated. This hyperactivation of microglia initiates inflammatory neurotoxicity, a process intimately associated with the loss of neuronal dendritic spines [17,18,19,20]. Among the various mechanisms that drive microglial activation, mitochondria [21,22,23]—serving as the primary energy source for these cells—are of critical importance. mtROS, which are essential byproducts of mitochondrial energy metabolism, can induce microglial activation through pathways such as the activation of NF-κB signaling and the opening of the mitochondrial permeability transition pore (mPTP) [24,25,26,27,28,29]. This process exacerbates inflammatory neurotoxicity and contributes to dendritic spine loss. Consequently, we hypothesize that hypoxia induces microglial activation via the generation of mtROS, leading to neurotoxicity-mediated dendritic spine loss and ultimately impairing higher-order brain functions. Furthermore, considering the central role of mtROS in microglial activation, the development of targeted therapeutics may protect neuronal structure and function following hypoxic exposure, thereby mitigating cognitive deficits.

Recent serial investigations have shown that supplementation with NR significantly enhances the activity of mitochondrial complex I, optimizes energy metabolism, and improves mitochondrial morphology and function [30]. Concurrently, NR intervention reduces mtROS levels in senescent cells, subsequently restoring the activity of mitochondrial sirtuin 3 (SIRT3) and activating antioxidant enzymatic pathways, such as superoxide dismutase 2 (SOD2) [31,32]. These effects collectively contribute to mitochondrial protection and exhibit anti-aging properties. In addition, NR and its metabolic derivatives can also improve ovarian function [33], reduce pulmonary inflammation [34] and the progression of neuroinflammation in neurodegenerative disease AD [13].

Given the capacity of NR to inhibit mtROS and the critical role of mtROS in triggering microglial activation, we propose the hypothesis that NR may suppress microglial activation by reducing intracellular mtROS levels. This reduction could potentially attenuate inflammatory neurotoxicity, mitigate neuronal dendritic spine loss, and ultimately enhance cognitive function.

## 2. Materials and Methods

### 2.1. Materials

Fetal bovine serum (13011-8611) was procured from Tianhang Biotechnology Company (Zhejiang, China). The DMEM medium (C11995500BT) was sourced from Gibco (Carlsbad, CA, USA). Biosharp Life Sciences (Anhui, China) supplied the 4% paraformaldehyde (BL539A). Electrophoresis buffer (P0014D), electroporation buffer (P0021B), enhanced adenosine triphosphate (ATP) detection kit (S0027), DAPI anti-fluorescence quenching mounting agent (P0131-5 ml), MitoSOX detection kit (S0061S), and the hematoxylin and eosin staining kit (C01050S) were all acquired from Beyotime Biotechnology Company (Shanghai, China). The BCA protein quantification kit (23225) was obtained from Thermo (Massachusetts, MA, USA). Phosphate-buffered saline (PBS) (MI00625) and ECL chemiluminescence reagent (MI00607A) were supplied by MiShu Biotechnology Company (Shaanxi, China). Rabbit anti-Iba-1 antibody (10904-1-AP), rabbit anti-β-actin antibody (81115-1-RR), rabbit anti-PSD95 antibody (30255-1-AP), rabbit anti-Snap25 antibody (14903-1-AP), and goat anti-rabbit secondary antibody (SA00001-2) were all procured from proteintech (Rosemont, IL, USA). The mouse anti-CD68 antibody (ab201973) was obtained from Abcam (Cambridge, UK). Fluorescent rabbit red secondary antibody (8889S) and fluorescent mouse green secondary antibody (4408S) were purchased from Cell Signaling Technology (Danvers, MA, USA). Isopropanol (67-63-0) and methanol (67-56-1) were procured from Fu Yu Chemical Company (Shandong, China). Diethyl pyrocarbonate (DEPC) (R1600) was sourced from Solarbio (Beijing, China). The FD Rapid GolgiStain™ Kit was acquired from FD Neurotechnologies (Columbia, MD, USA). The reverse transcription kit and the real-time fluorescence quantitative PCR kit (RR820A) were obtained from TAKARA (Shiga, Japan). The Morris Water Maze and Novel Object Recognition were purchased from Shanghai Xinran Information Technology (Shanghai, China). The Forma Series II CO_2_ cell incubator was acquired from Thermo Fisher Scientific (San Francisco, CA, USA). The Western blot electrophoresis and transfer system were procured from Bio-Rad Laboratories (Hercules, CA, USA). The QuantStudio 7 real-time fluorescence quantitative PCR instrument was obtained from Thermo Fisher Scientific. The Infinite F200 enzyme reader was sourced from Tecan Group Ltd (Hombrechtikon, Switzerland). The Mini-PROTEAN protein electrophoresis system was purchased from Bio-Rad Laboratories. The fluorescence microscope was acquired from Olympus Corporation (Tokyo, Japan). The JEM-1230 transmission electron microscope was sourced from JEOL Ltd (Tokyo, Japan).

### 2.2. Methods

#### 2.2.1. The Animal Model and Treatment

Eight-week-old male C57BL/6 mice were procured from the Animal Experiment Center of the Fourth Military Medical University and acclimatized in a pathogen-free animal facility for four days. The mice were maintained under controlled conditions with regulated ventilation, temperature, and humidity, adhering to a 12 h light-dark cycle. They were provided with unrestricted access to water and food. All experimental protocols were in strict compliance with the guidelines set forth by the Animal Experiment Ethics Committee of the Fourth Military Medical University (SCXK (Shaanxi) 2024-0745). Following acclimatization, the mice were randomly assigned to four experimental groups: normoxia with normal saline, normoxia with NR treatment, hypoxia with normal saline, and hypoxia with NR treatment, with each group comprising six mice. A hypoxic mouse model was established using an environmental simulation chamber to mimic the low-oxygen conditions at an altitude of 6000 m [35]. Daily dosage of 400 mg/kg of NR was administered for two weeks through intraperitoneal injection. In contrast, control group mice were maintained in the standard animal breeding facility. Two weeks after low-oxygen exposure, mice from the control group and the low-oxygen group at corresponding time points were simultaneously taken out for behavioral index detection and tissue sampling for subsequent experiments.

#### 2.2.2. Cell Culture and Treatment

BV2 and HT22 cell lines were generously provided by Professor Wang Yazhou from the Department of Neurobiology at the Fourth Military Medical University. These cells were maintained in a DMEM basal medium supplemented with 4.5 g/L D-glucose and 10% FBS at 37 °C in a humidified atmosphere containing 5% CO_2_. For the co-culture experiments, BV2 cells were seeded at appropriate densities into 10 mm cell culture dishes and subjected to either normoxic conditions or 1% O_2_ in Don Whitley Scientific hypoxic workstations. Following a 48 h exposure period, the BV2 cell culture medium was harvested and centrifuged at 1000 rpm for 5 min to remove cellular debris, with the supernatant subsequently collected. This BV2 cell-derived supernatant was then applied to HT22 cells under normoxic conditions, and the cells were incubated at 37 °C with 5% CO_2_ for 24 h. 0.5 mM NR was used in conditional co-culture model. Post-treatment, HT22 cell proteins were extracted and stored at −80 °C for future experimental analyses.

#### 2.2.3. Morris Water Maze

Two hours prior to the commencement of the experiment, the mice were introduced to the behavioral testing room to acclimate to the environment. Distinctively colored and shaped symbols were marked on the walls of the four quadrants of the water maze. An underwater platform was positioned in the first quadrant of the water maze, submerged 1 cm below the water surface. Titanium dioxide white dye was added to the pool to render the water a milky white color, and the water temperature was maintained at 21–22 °C. The mice were sequentially introduced into the water maze, starting from the first quadrant and proceeding to the fourth, with each learning session lasting for 1 min. If a mouse successfully located the platform within the allotted time, it was permitted to remain on the platform for 15 s before being removed. Conversely, if a mouse failed to find the platform within 1 min, it was guided to the platform and allowed to remain for 15 s before removal. Each mouse participated in a daily learning session within each of the four quadrants, a protocol that was maintained over a period of five consecutive days. During the testing phase, the submerged platform was removed, and the mice were introduced into the water maze from the third quadrant, which is diametrically opposite to the initial quadrant of the platform. The parameters recorded included the average swimming speed of the mice over a one-minute interval, the latency to escape the platform in the water maze, the frequency of crossings over the original platform location, and both the duration and distance of swimming within the target quadrant, identified as the first quadrant.

#### 2.2.4. Novel Object Recognition

Prior to the commencement of the experiment, the mice were introduced to the behavioral testing laboratory to acclimate to the environment. The interior of the novel object recognition apparatus was sanitized using alcohol wipes, and items from Group A and Group B were positioned at predetermined locations to ensure that the colors and shapes of the items were comparable and devoid of any distinctive odors. During the learning phase of the experiment, the mice were placed in the apparatus and permitted to explore the objects freely for a duration of five minutes. Following the removal of the mice, the interior of the apparatus was again sanitized with alcohol wipes. In the testing phase, the items from Group A were substituted with those from Group C, and the mice were reintroduced to the apparatus for exploration. The exploration time and frequency for Group B (familiar objects) and Group C (novel objects) were meticulously recorded during the testing phase. Subsequently, the novel object recognition index was calculated to assess alterations in the cognitive functions of the mice.

#### 2.2.5. Hematoxylin and Eosin Staining (HE)

Mice under deep anesthesia were subjected to perfusion with pre-cooled normal saline followed by a 4% paraformaldehyde solution. Subsequently, the entire brain tissue was harvested and fixed in a 4% paraformaldehyde solution for a duration of 24 h. The brain tissue specimens were then embedded in paraffin, and serial coronal sections of the hippocampal region, each with a thickness of 12 μm, were prepared in accordance with the mouse brain atlas. To remove paraffin, the sections were immersed in fresh xylene for 10 min, a process repeated twice. This was followed by immersion in a series of ethanol solutions of varying concentrations (absolute ethanol, 95% ethanol, 85% ethanol, and 75% ethanol) for 5 min each to achieve gradient hydration. The sections were initially stained with hematoxylin for 10 min, then placed in a slide chamber and rinsed with running water for 5 min. Subsequently, the sections were immersed in a hydrochloric acid ethanol differentiation solution for 10 s, followed by immersion in a re-blotting solution for 3 min. The sections were again placed in a slide chamber and rinsed with running water for 2 min. Finally, the sections underwent gradient dehydration by sequential immersion in 75% ethanol, 85% ethanol, and 95% ethanol for 5 min each. The tissue sections underwent a staining process involving immersion in eosin dye for a duration of 5 min. Subsequently, the sections were immersed in anhydrous ethanol for 2 min to remove any excess eosin dye. For continuous clearing treatment, the sections were then immersed twice in fresh xylene solvent, each for a period of 5 min. Finally, the sections were sealed using neutral gum and cover glass, and subsequently examined under a microscope.

#### 2.2.6. Golgi Staining

Induce deep anesthesia in mice and excise the entire brain tissue. Initially, submerge the tissue blocks in the immersion solution and incubate them at 4 °C in darkness for 14 days, refreshing the immersion solution every 3 days. Subsequently, transfer the tissue blocks to a protective solution and stabilize them at 4 °C in darkness for 72 h. Following stabilization, employ a vibrating microtome to section the tissue, and then immerse the sections in a developing and fixing solution (1:1 ratio). Stain the sections at room temperature in darkness for 60 min. Upon completion of the staining process, transfer the sections to a termination solution for soaking, and rinse off any residual developing solution with deionized water. Finally, subject the sections to a gradient dehydration process by immersing them sequentially in 75% ethanol, 85% ethanol, 95% ethanol, and absolute ethanol for 5 min each. Subsequently, immerse the sections in fresh xylene solvent for 5 min for clearing. Mount the sections using neutral gum and a cover glass, and examine them under a microscope.

#### 2.2.7. Immunohistochemical Fluorescence Staining

The frozen sections were allowed to air dry at ambient temperature. Subsequently, the sections underwent three washes with 1× PBS, each lasting 5 min. A permeabilization solution containing 0.3% Triton X-100 was then applied to the sections at room temperature for a duration of 30 min. Following permeabilization, a blocking step was performed using a 5% BSA solution at room temperature for 1 h. The sections were then incubated with primary antibodies against Iba-1 and CD68 at 4 °C overnight. On the following day, fluorescence-conjugated secondary antibodies were applied under light-protected conditions and incubated at room temperature for 1 h. Excess liquid was carefully removed using filter paper. The sections were subsequently treated with a fluorescence quenching inhibitor containing DAPI and mounted with a coverslip. The samples were examined and imaged using a laser fluorescence microscope, and the fluorescence images were analyzed and subjected to statistical processing using ImageJ software V1.8.0.112.

#### 2.2.8. Transmission Electron Microscope (TEM)

The hippocampal tissue specimens were initially fixed in 2.5% glutaraldehyde at 4 °C for a duration of 24 h, followed by a secondary fixation with 1% osmium tetroxide for 1 h. Subsequently, the samples underwent a graded dehydration process utilizing 100% absolute ethanol, with each step of dehydration maintained for 15 min. This was followed by a final dehydration step using 100% acetone for 25 min. Post-dehydration, the tissue samples were embedded and placed in a constant-temperature drying oven set at 60 °C for 48 h. The desiccated tissue samples were then sectioned into ultrathin slices measuring 60–80 nm using an ultramicrotome. These sections were stained with 1% uranyl acetate for 10 min, rinsed with PBS, and subsequently examined using a JEM-1230 transmission electron microscope for image acquisition and analysis.

#### 2.2.9. Real-Time Fluorescence Quantitative PCR (RT-PCR)

Following treatment of the animal hippocampal tissue and cell samples with 1 mL of Trizol, the samples underwent extraction and purification utilizing trichloromethane, isopropanol, and 75% anhydrous ethanol for subsequent quantitative analysis. Complementary DNA (cDNA) synthesis was then performed using the TB Green^®^ Premix Ex Taq™ II (RR820A) reverse transcription kit. Quantitative detection of target mRNA expression levels in each group of cells or tissues was conducted using real-time fluorescent quantitative PCR detection kits. Data analysis was carried out employing the 2^−∆∆Ct^ method. The primer sequences utilized in this study are detailed in Table 1.

#### 2.2.10. Western Blot

Tissue specimens were homogenized in ice-cold lysis buffer (containing 0.1% SDS, 1× protease inhibitor cocktail, and PMSF) using sequential mechanical homogenization and sonication. The lysates were centrifuged at 12,000× *g* for 15 min at 4 °C, followed by transfer of the supernatant to fresh microcentrifuge tubes. Protein concentration was quantified via BCA assay. Subsequently, 5× Laemmli sample buffer was added to the samples, which were then denatured at 95 °C in a metal bath for 10 min prior to storage at −80 °C or immediate electrophoresis. Select an appropriate percentage separation gel according to the molecular weight of the target protein. The electrophoresis should be conducted at 80 V for 30 min, followed by 120 V until the target protein is adequately separated. Prepare a 1× membrane transfer buffer in accordance with the reagent instructions, and carry out the membrane transfer at 250 mA. Subsequent to the membrane transfer, incubate the PVDF membrane at room temperature for 1 h to achieve blocking. Then, rotate and incubate the membrane with the primary antibody at 4 °C overnight. Upon completion of the primary antibody incubation, rotate and incubate with the secondary antibody at room temperature on a shaker for 1 h. After the secondary antibody incubation, place the PVDF membrane in a chemiluminescence imaging instrument, apply the ECL luminescent reagent evenly, capture the image, and utilize ImageJ software for quantitative analysis of the protein bands.

#### 2.2.11. Determination of NMN Concentrations

To prepare mouse hippocampal tissue for analysis, 500 μL of 1× PBS was first added to the sample, followed by thorough homogenization using a mechanical homogenizer. Subsequently, the homogenate was centrifuged at 3000× *g* for 20 min at 4 °C, after which the supernatant was collected. Blank and sample wells were then designated in a 96-well plate. Next, 10 μL of supernatant was aliquoted into sample wells and 40 μL diluent was added. Following this, 100 μL enzyme-conjugated reagent was added to all wells except blanks. The plate was subsequently incubated at 37 °C for 60 min in a temperature-controlled chamber. Meanwhile, wash buffer was prepared by diluting concentrate with deionized water. Upon completion of incubation, wells were filled with wash solution and allowed to stand for 30 s before decanting. Thereafter, 50 μL each of chromogenic substrates A and B were added to all wells, and the plate was incubated at 37 °C for 15 min protected from light. Reactions were then terminated by adding 50 μL stop solution per well. Finally, the microplate reader was zeroed using blank wells within 15 min of termination, optical density at 450 nm was measured, and NMN concentrations were calculated using the standard curve.

#### 2.2.12. Determination of NAD+ Concentrations

The experimental procedure commences with the collection of mouse hippocampal tissue, to which 500 μL of ice-cold 1× PBS was added. Subsequently, the sample was thoroughly homogenized using a mechanical homogenizer at 1500 rpm for 5 min. Following homogenization, the mixture was centrifuged at 12,000× *g* for 15 min at 4 °C, after which the supernatant was discarded and the pellet resuspended in 100 μL lysis buffer. The resuspended sample was then incubated at 37 °C for 15 min. Next, NAD+ extraction solution and NAD+ control solution were introduced to the lysate, with the incubation continued at 37 °C for an additional 15 min period. During this step, 15 μL of corresponding assay buffer was added per 15 μL of sample. Following NAD+ extraction, the reaction was terminated by adding stop buffer. The processed samples then received 75 μL of NAD+ reaction mixture and undergone room temperature incubation for 1 h. Finally, fluorescence measurements were performed using a microplate reader with excitation at 540 nm and emission at 590 nm. The recorded fluorescence values were subsequently compared against a standardized calibration curve to quantify NAD+ concentration.

#### 2.2.13. Measurement of Mitochondrial ATP Levels

For cellular specimens, lysis was followed by centrifugation at 12,000× *g* for 5 min at 4 °C, after which the supernatant was collected for subsequent analysis. Regarding hippocampal tissue samples, lysis buffer was first introduced at a ratio of 100–200 μL per 20 mg tissue. Subsequently, thorough homogenization was performed using a glass homogenizer or equivalent mechanical disruption system to ensure complete tissue dissociation. Following homogenization, the lysate undergone centrifugation at 12,000× *g* for 5 min at 4 °C, and the resulting supernatant was collected. For ATP quantification, an appropriate volume of sample supernatant was then aliquoted alongside standards and detection reagent into microplate wells. After gentle mixing by pipetting, measurements were conducted using a microplate reader. Finally, ATP concentrations were calculated by extrapolating absorbance values against the standard curve.

#### 2.2.14. Measurement of MitoSOX

The MitoSOX Red fluorescent probe is capable of detecting superoxide within mitochondria and emits a robust fluorescent signal. Following the kit’s protocol, the MitoSOX staining working solution should be prepared and applied to the cells. Each cell group should be washed once with PBS, followed by the addition of 1 mL of the MitoSOX Red staining working solution. The cells should then be incubated at 37 °C in a cell culture incubator for 15 min. Subsequently, wash the cells three times with PBS buffer, add the cell culture medium, and measure the fluorescence intensity of each group using the Tecan Infinite F200 multi-functional microplate reader.

#### 2.2.15. Statistical Analysis

Each experiment was conducted a minimum of three times. Statistical analyses and graphical representations were carried out utilizing GraphPad Prism version 8.0.2. The experimental data are presented as the mean ± standard deviation (x ± s). For comparisons between two groups, the *t*-test was employed, while one-way ANOVA was utilized for comparisons among multiple groups. *p* < 0.05 was considered indicative of a statistically significant difference.

## 3. Results

### 3.1. Hypoxic Impairment of Learning and Memory Functions in Mice

**Figure 1:**
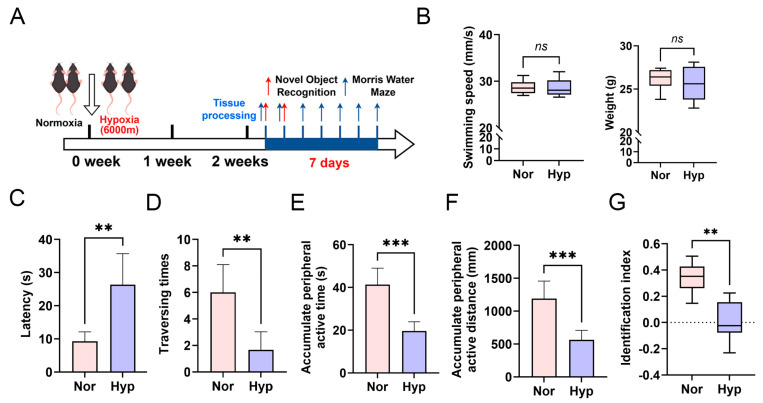
Hypoxic impairment of learning and memory functions in mice. (**A**) Illustration of the 6000 m low-oxygen exposure protocol for 8-week-old male C57 mice. (**B**) Analysis of swimming speed and body weight in mice following low-oxygen exposure. (**C**) Assessment of escape latency in the Morris water maze experiment. (**D**) Evaluation of platform crossing frequency in the Morris water maze spatial exploration experiment. (**E**) Measurement of time spent in the target quadrant during the Morris water maze spatial exploration experiment. (**F**) Quantification of distance traveled within the target quadrant in the Morris water maze spatial exploration experiment. (**G**) Assessment of the novel object recognition index in the novel object recognition experiment. *ns p* > 0.05; ** *p* < 0.01; *** *p* < 0.001.

The research team successfully developed a mouse model subjected to an altitude of 6000 m for a duration of two weeks (Figure 1A). The results indicated that, in comparison to the normoxic control group, there were no significant changes in body weight or swimming speed in the hypoxic exposure group (Figure 1B), suggesting that high-altitude hypoxic exposure did not impact the motor abilities of the mice. However, further behavioral assessments revealed that, relative to the normoxic control group, mice exposed to hypoxia exhibited a significantly increased escape latency in the Morris water maze (Figure 1C), a significant reduction in platform crossings (Figure 1D), decreased duration spent in the target quadrant (Figure 1E), reduced distance traveled in the target quadrant (Figure 1F), and a significantly lower novel object recognition index (Figure 1G). These findings collectively suggest that high-altitude hypoxic exposure can impair learning and memory functions in mice.

### 3.2. Hypoxia Impairs Dendritic Spine Plasticity in Hippocampal CA1 Neurons and Activates Microglia in Mice

**Figure 2:**
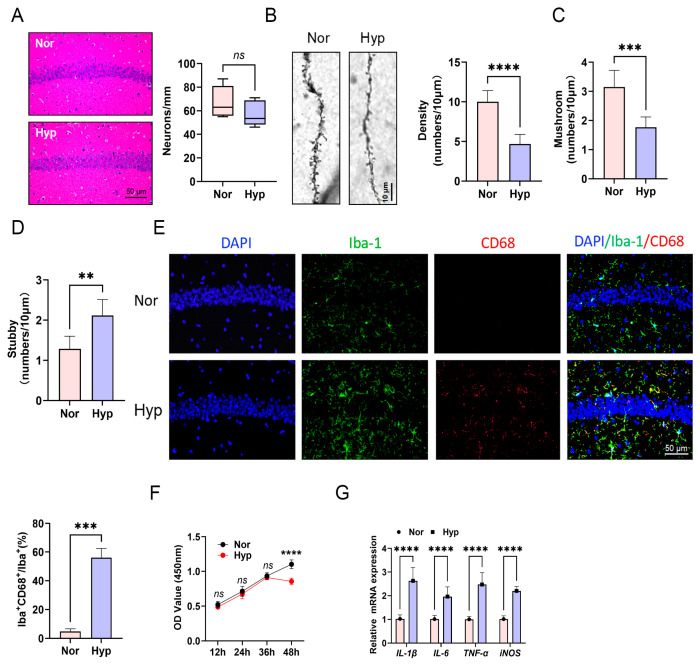
Hypoxia impairs dendritic spine plasticity in hippocampal CA1 neurons and activates microglia in mice. (**A**) HE staining was utilized to quantify the neuronal population in the CA1 region of the hippocampus. (**B**) Golgi staining was applied to assess the density of dendritic spines in the CA1 region of the hippocampus. (**C**) Golgi staining facilitated the examination of Mushroom-shaped dendritic spine numbers. (**D**) Golgi staining facilitated the examination of Stubby-shaped dendritic spine numbers. (**E**) Immunohistochemical fluorescence staining was conducted to determine the proportion of Iba^+^CD68^+^/Iba^+^ microglial cells. (**F**) The CCK8 assay was employed to measure the optical density (OD) values of BV2 cells at 12, 24, 36, and 48 h. (**G**) RT-PCR was used to quantify the expression levels of *IL-1β*, *IL-6*, *TNF-α*, and *iNOS* mRNA in BV2 cells at 36 h. *ns p* > 0.05; ** *p* < 0.01; *** *p* < 0.001; **** *p* < 0.0001.

The functions of learning and memory in mice are intricately linked to neuronal density and dendritic spine plasticity within the hippocampal CA1 region. HE staining indicated a slight reduction in neuronal density in the hippocampal CA1 area of mice subjected to hypoxic conditions compared to those maintained under normoxic conditions (Figure 2A). No significant loss of neuronal numbers was observed after exposure to low oxygen, thereby necessitating further investigation into the impact of hypoxia on dendritic spine plasticity. Golgi staining revealed a significant decrease in dendritic spine density in hippocampal CA1 neurons following hypoxic exposure (Figure 2B). Subtype analysis identified a pronounced reduction in mushroom-type spines (Figure 2C) alongside an increase in stubby-type spines (Figure 2D). Concurrently, immunohistochemical fluorescence staining demonstrated a significant increase in the number of CD68^+^ and Iba-1^+^ double-labeling staining cells in the CA1 region of hypoxic-exposed mice (Figure 2E). In vitro experiments showed a substantial reduction in BV2 cells viability after 48 h of hypoxic exposure, whereas no significant differences were observed at 12 h, 24 h or 36 h time points (Figure 2F). Consequently, a 36 h hypoxia exposure was selected for subsequent in vitro modeling. RT-PCR analysis showed significantly upregulated mRNA levels of *IL-1β*, *IL-6*, *TNF-α*, and *iNOS* in BV2 cells following 36 h hypoxia compared to normoxic controls (Figure 2G). Collectively, these findings demonstrate that hypoxic exposure impairs dendritic spine plasticity in hippocampal CA1 neurons and activates microglia.

### 3.3. NR Treatment Preserves Learning and Memory Functions in Mice

**Figure 3:**
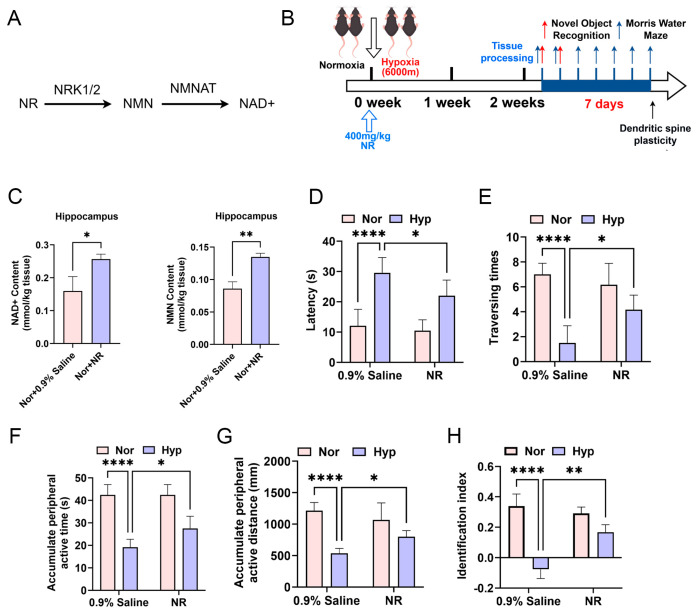
NR treatment preserves learning and memory functions in mice. (**A**) The diagram of NR is metabolized into NMN, subsequently leading to the production of NAD+. (**B**) A model diagram illustrates the intervention treatment involving a 400 mg/kg intravenous injection of NR administered to mice subjected to 6000 m low-oxygen exposure. (**C**) The concentrations of NAD+ and NMN in the hippocampal tissues of mice from each group following the 400 mg/kg NR intervention. (**D**) Assessment of escape latency in the Morris water maze experiment. (**E**) Evaluation of platform crossing frequency in the Morris water maze spatial exploration experiment. (**F**) Measurement of time spent in the target quadrant during the Morris water maze spatial exploration experiment. (**G**) Quantification of distance traveled within the target quadrant in the Morris water maze spatial exploration experiment. (**H**) Assessment of the novel object recognition index in the novel object recognition experiment. * *p* < 0.05; ** *p* < 0.01; **** *p* < 0.0001.

Current evidence suggests that NR is converted to nicotinamide mononucleotide (NMN) through catalysis by nicotinamide riboside kinases 1 and 2 (NRK1/2), thereby enhancing nicotinamide adenine dinucleotide (NAD+) biosynthesis [36] (Figure 3A). In this study, mice were administered daily dose of 400 mg/kg NR intravenously over a two-week period (Figure 3B). Subsequent analyses indicated a significant increase in hippocampal NMN and NAD^+^ concentrations (Figure 3C). Behavioral evaluations revealed that, compared to the hypoxia-exposed control group, NR-treated hypoxic mice demonstrated: a significantly reduced escape latency in the Morris water maze (Figure 3D), an increased number of platform crossings (Figure 3E), prolonged time spent in the target quadrant (Figure 3F), an extended path length within the target quadrant (Figure 3G), and an improved novel object recognition index (Figure 3H). Collectively, these findings indicate that NR intervention effectively ameliorates learning and memory deficits in mice.

### 3.4. NR Mitigates Hypoxia-Induced Dendritic Spine Plasticity Impairment

**Figure 4:**
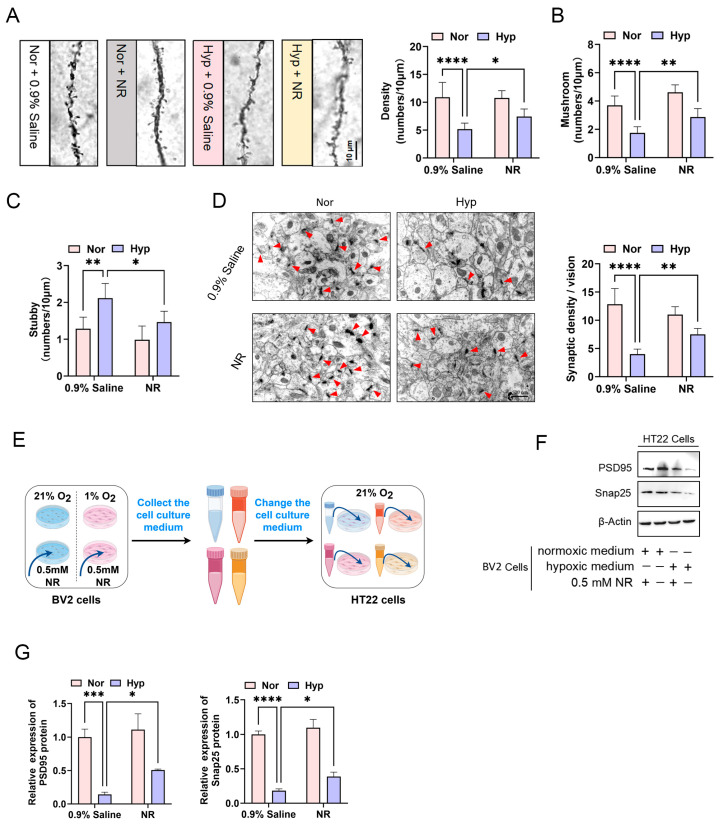
NR mitigates hypoxia-induced dendritic spine plasticity impairment. (**A**) Golgi staining was employed to assess the density of dendritic spines in the CA1 region of hippocampal neurons across different groups of mice following NR intervention administration. (**B**) Golgi staining was utilized to quantify the number of Mushroom-type dendritic spines. (**C**) Golgi staining was applied to evaluate the number of Stubby-type dendritic spines. (**D**) TEM was employed to examine the synaptic density of hippocampal neurons in the CA1 region. Red arrow represents the synapse of between neurons. (**E**) A schematic diagram illustrates the conditional co-culture of HT22 neuronal cells and BV2 microglial cells. (**F**) Western blot analysis was conducted to detect the expression levels of PSD95 and Snap25 proteins in HT22 neuronal cells. (**G**) Gray-scale analysis was performed to determine the relative expression levels of PSD95 and Snap25 proteins. * *p* < 0.05; ** *p* < 0.01; *** *p* < 0.001; **** *p* < 0.0001. Original Western blot images can be found in Supplementary File S1.

Subsequent investigations demonstrated that, in comparison to the group exposed solely to hypoxia, mice subjected to a combination of hypoxia and NR treatment exhibited a significant increase in dendritic spine density within hippocampal CA1 neurons (Figure 4A), an elevation in the number of mushroom-type spines (Figure 4B), and a reduction in the number of stubby-type spines (Figure 4C). TEM revealed a pronounced decrease in synaptic density and number in mice exposed to hypoxia compared to normoxic controls, with NR intervention partially ameliorating these deficits (Figure 4D). To further investigate the association between microglial activation and synaptic loss, a co-culture system comprising BV2 microglia and HT22 neuronal cells was established under hypoxic conditions (Figure 4E). Western blot analysis indicated a significant reduction in the levels of PSD95 and Snap25 proteins in HT22 cells treated with conditioned medium from hypoxia-exposed BV2 microglia, as opposed to those treated with control medium (Figure 4F,G). Importantly, administration of 0.5 mM NR restored the expression of these synaptic proteins relative to the hypoxia-only group (Figure 4F,G). In summary, these findings indicate that NR intervention alleviates hypoxia-induced deficits in dendritic spine plasticity.

### 3.5. NR Attenuates Neurotoxic Damage Mediated by Inflammatory Factors Through Suppression of mtROS Production and Microglial Activation

**Figure 5:**
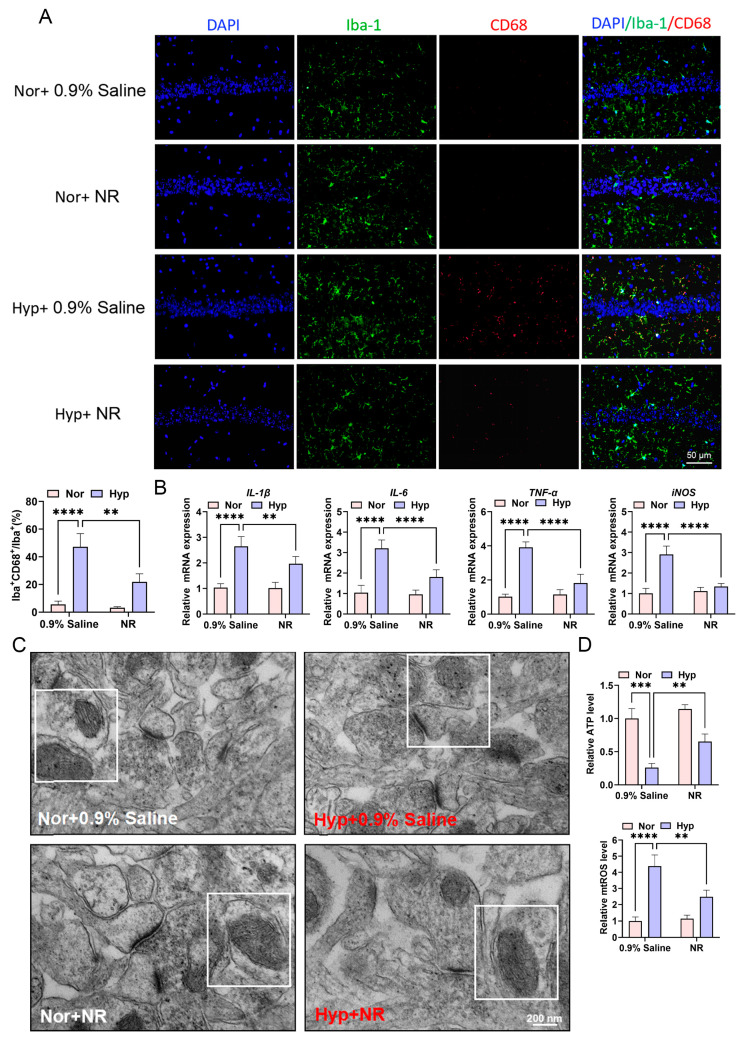
NR attenuates neurotoxic damage mediated by inflammatory factors through suppression of mtROS production and microglial activation. (**A**) Immunohistochemical fluorescence staining was used to observe the percentage of CD68^+^Iba^+^/Iba^+^ microglia cells in the CA1 region of the hippocampus of mice in each group after NR intervention administration. (**B**) RT-PCR was used to detect the expression levels of *IL-1β*, *IL-6*, *TNF-α*, and *iNOS* mRNA in BV2 microglial cells of each group after 0.5 mM NR intervention. (**C**) TEM was used to observe the morphological changes in mitochondrial membranes and mitochondrial cristae in the CA1 region of the hippocampus of mice in each group after NR intervention administration. The image within the white box represents the typical morphology of a mitochondrion. (**D**) The levels of ATP and mtROS in BV2 cells of each group after 10 mM NR intervention. ** *p* < 0.01; *** *p* < 0.001; **** *p* < 0.0001.

Immunofluorescence analysis demonstrated a significant reduction in the percentage of CD68^+^Iba-1^+^/Iba-1^+^ cells within the hippocampal CA1 region of mice exposed to hypoxia and concurrently treated with NR, as opposed to those subjected solely to hypoxia (Figure 5A). RT-PCR revealed a substantial decrease in the expression levels of pro-inflammatory cytokines *IL-1β*, *IL-6*, *TNF-α*, and the *iNOS* in BV2 cells under hypoxic conditions with a 0.5 mM NR intervention, compared to hypoxia alone (Figure 5B). Recent studies suggest that elevated mtROS levels are a critical factor in neurodegenerative diseases, with mtROS-induced microglial activation playing a significant role. TEM indicated compromised mitochondrial cristae and membrane integrity in hippocampal cells exposed to hypoxia, in contrast to normoxic controls, while NR intervention mitigated these ultrastructural abnormalities (Figure 5C). Additional assays revealed a significant decrease in ATP levels and an increase in mtROS levels in BV2 cells following hypoxic exposure. Importantly, NR co-treatment counteracted these metabolic disturbances, resulting in elevated ATP levels and reduced mtROS production compared to conditions of hypoxia alone (Figure 5D). Collectively, these findings establish that NR intervention attenuates inflammatory factor-mediated neurotoxic damage by suppressing mtROS generation and subsequent microglial activation.

## 4. Discussion

The hypoxic conditions encountered at high altitudes represent a significant physiological stressor and have emerged as a focal point of research within the domain of high-altitude medicine, primarily due to their impact on central nervous system functions. The brain is particularly sensitive to fluctuations in environmental oxygen levels, making it especially vulnerable to the effects of high-altitude hypoxia. Synapses are closely linked to disruptions in higher-order brain functions. Despite this understanding, existing clinical pharmacotherapies targeting neuronal synaptic plasticity remain limited by suboptimal efficacy. In this study, we developed a hypoxic model using mice exposed to an altitude of 6000 m for a duration of two weeks. Utilizing a combination of behavioral, morphological, and molecular biological methodologies, we systematically investigated the effects of high-altitude hypoxia on the learning and memory functions of mice, elucidating potential underlying mechanisms. Furthermore, we evaluated the neuroprotective effects of NR intervention, providing experimental evidence for understanding the pathophysiological process of hypoxic cognitive impairment and developing targeted prevention and treatment strategies.

This study demonstrates that exposure to hypoxic conditions at high altitudes can adversely affect the cognitive functions related to learning and memory in mice. This is evidenced by an increased latency in locating the escape platform in the Morris water maze, a marked reduction in the frequency of platform crossings, diminished time and distance spent in the target quadrant, and a significant decrease in the novel object recognition index. Subsequent investigations revealed that hypoxia notably diminishes dendritic spine density in the CA1 region of the hippocampus, resulting in a reduction in mushroom dendritic spines associated with long-term memory, an increase in stubby dendritic spines, and the activation of microglia, which release pro-inflammatory cytokines such as IL-1β, IL-6, and TNF-α. This neuroinflammatory response induces neurotoxic damage and further impairs the plasticity of neuronal dendritic spines.

The accumulation of mtROS can activate the NF-κB signaling pathway, thereby enhancing microglial activation and inducing the opening of the mPTP, which in turn impairs mitochondrial function and exacerbates inflammatory neurotoxicity [24,25,26,27,28,29]. When mtROS levels increase, IκB is phosphorylated and subsequently degraded, leading to the release of NF-κB [37]. This transcription factor translocates into the nucleus and binds to specific sequences within the promoter regions of target genes, resulting in the upregulation of various inflammatory cytokines, such as IL-1β, IL-6, and chemokines. This process intensifies the inflammatory response and perpetuates microglial activation. Additionally, mtROS can directly induce the opening of the mPTP [38]. The excessive opening of the mPTP detrimentally affects mitochondrial structure and function, resulting in the collapse of mitochondrial membrane potential and diminished ATP production. This cascade further disrupts immune homeostasis within the CNS and exacerbates inflammatory neurotoxicity, ultimately promoting the loss of neuronal dendritic spines. Recent studies have demonstrated that NR not only mitigates electron leakage by enhancing the activity of complex I [39] but also restores mitochondrial SIRT3 activity [40]. Additionally, NR activates antioxidant enzymes such as SOD2, as evidenced by experiments on aged mice, which show a reduction in mtROS and confer mitochondrial protection and anti-aging benefits via the SIRT3-SOD2 pathway [31]. This study has further confirmed that high-altitude hypoxia triggers the activation of microglia in the CA1 region of the hippocampus, mediating neuroinflammatory responses that lead to impaired dendritic spine plasticity and consequent learning and memory deficits in mice. Conversely, NR treatment enhances NAD+ production and improves mitochondrial energy metabolism, thereby inhibiting mtROS generation, reducing neuroinflammatory toxicity, and ameliorating dendritic spine plasticity.

This experiment represents the inaugural proposal of utilizing NR intervention to mitigate the inflammatory neurotoxic effects of microglia, thereby ameliorating the cognitive impairments induced by hypoxic exposure in mice. Nonetheless, this study is subject to certain limitations. Primarily, while acute hypoxia exposure typically results in immediate brain injury, NR intervention is predominantly associated with long-term and chronic therapeutic outcomes. Consequently, the application of NR in the treatment or prevention of acute hypoxic brain injury remains unresolved. Furthermore, the investigation into the molecular mechanisms by which NR attenuates microglial neurotoxicity is relatively underdeveloped. Additional research is warranted to elucidate the protective effects of NR on mitochondrial oxidative stress and structural integrity, as well as its regulatory mechanisms within microglia. The protective effects of NR on mitochondrial oxidative stress and structural integrity, along with its regulatory mechanisms in microglia, warrant further investigation. In vivo studies have demonstrated that NR intervention significantly enhances mitochondrial membrane stability following hypoxia exposure and diminishes mitochondrial mtROS levels. This suggests that NR intervention may inhibit microglial NF-κB inflammatory signaling, potentially by reducing mtDNA leakage and decreasing mtROS production. Furthermore, the mPTP functions as a non-specific channel within the inner mitochondrial membrane, and NR intervention has been shown to preserve mitochondrial membrane stability post-hypoxia, indicating its role in maintaining the structural integrity of mPTP. Additionally, mtROS can directly influence mPTP by inducing its opening, and the reduction in mtROS levels through NR intervention implies that the NR-mtROS axis may be pivotal in regulating the normal function of mPTP and its associated downstream signaling pathways. Future research should integrate gene editing and omics technologies to delve deeper into the relationship and mechanisms linking microglial inflammatory activation with higher-order brain functions. Building on this study, such research aims to establish a robust theoretical foundation and develop effective strategies for preventing and treating brain function impairment following hypoxic exposure.

## 5. Conclusions

Our research demonstrates that hypoxia can trigger inflammatory neurotoxicity in microglia via mtROS, leading to synaptic plasticity impairment in the CA1 region of the hippocampus and resulting in learning and memory deficits in mice. NR, a precursor of NAD+, was found to effectively reduce mtROS levels in microglia following hypoxic exposure, mitigate the loss of neuronal dendritic spines caused by microglial neurotoxicity, and thereby confer neuroprotective effects. In conclusion, this study elucidates the pivotal role of mtROS in hypoxia-induced microglial neurotoxicity and suggests that NR, as an inhibitor of mtROS production, holds potential clinical value for the treatment of hypoxic encephalopathy.

## Figures and Tables

**Table 1:** Real-time qPCR primer sequences.

	Forward Primer	Reverse Primer
*IL-1β*	GCCCATCCTCTGTGACTCAT	AGGCCACAGGTATTTTGTCG
*TNF-α*	CGTCAGCCGATTTGCTATCT	CGGACTCCGCAAAGTCTAAG
*IL-6*	CCTTGAGGTTAGTGAACGTCA	CGCTCTCGTTTTCCCCATAATC
*iNOS*	ACCAAGTTCTCTTCGTTGAC	CTTCACAGAGAGGGTCACAG
*β-Actin*	GCTCCTCCTGAGCGCAAGTA	GCAGCTCAGTAACAGTCCGC

## Data Availability

The original contributions presented in this study are included in the article. Further inquiries can be directed to the corresponding author.

## Supplementary Materials

The following supporting information can be downloaded at: https://www.mdpi.com/article/10.3390/biom15101391/s1, File S1: Original Western blot images.
