# Supplementary figures and images for: Nicotinamide Riboside Alleviates the Neurotoxic Injury of Dendritic Spine Plasticity Mediated by Hypoxic Microglial Activation
Source: Biomolecules. 2025 Sep 30;15(10):1391. doi: 10.3390/biom15101391 (PMC12562574; doi:10.3390/biom15101391)

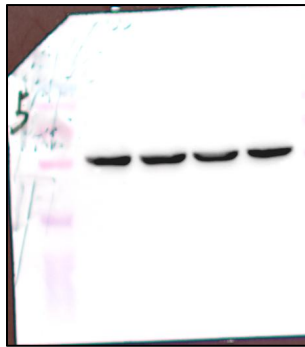

43kd  $\beta$ -Actin

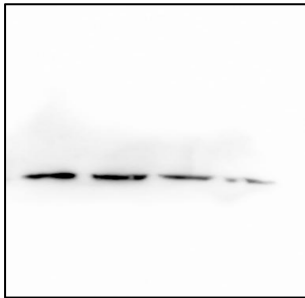

25kd Snap25

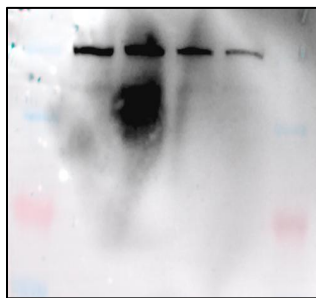

95kd PSD95

Supplement: Supplementary file 1 [file biomolecules-15-01391-s001.zip › biomolecules-3869657-supplementary.pdf]
